# Third Clinical Report on Continued Fever, Based on an Analysis of Sixty-Four Cases

**Published:** 1852-07

**Authors:** 


ART. VI. — *Third Clinical Report on Continued Fever, based on an Analysis of Sixty-four Cases.* By the EDITOR.

(Continued from page 31 and concluded.)

SECTION NINTH.

*Symptoms (exclusive of eruptions) referable to the Skin.*

Having given in the Second Report the symptoms relating to temperature, dryness, moisture and sweating, as noted in a considerable number of cases, individually, on successive days during the progress of the disease, I shall dispense, in the present Report, with these details. Were I to prepare similar tables (the data for which are before me) so far as I can judge from a general survey of the facts, the results would be materially the same. There would be nothing gained by such a repetition. I propose in this section to study the cases with reference to a single subject, which is one of interest' and importance. I refer to the occurrence of perspiration in the course of Continued Fever, its influence on the progress and issue of the disease, and its significance as a prognostic. The results disclosed by the former analyses led to conclusions at variance with ideas which are frequently, if not generally entertained by medical practitioners. These conclusions were, "that we are not warranted in predicating expectations of speedy convalescence, or of recovery, upon either of these symptoms (moisture and free perspiration) disconnected from other circumstances, nor do these results afford any grounds for supposing that to induce moisture or sweating by therapeutical means, will be likely to prove beneficial." Now, do the facts contained in the present collection of cases sustain these conclusions? In the former analyses it was found that moisture and sweating "were observed at different periods of the febrile career, in a large proportion of instances not preceding by a short space of time the date of convalescence. Moreover, these appearance were observed in nearly one-half of the cases which ended fatally." These were the considerations upon which were based the conclusions just stated. Let us see if, on examination of the cases in this collection, facts are found to correspond with those heretofore developed.

It will suffice to interrogate the histories respecting the presence of sensible perspiration without taking pains to state whether it consisted in moisture, or in free sweating.

Excluding fatal cases, of the *thirty-seven* remaining in the *Typhus* group, in *twenty-eight*, *moisture* of the surface or sweating more or less profuse, occurred either once, or several times, during the course of the disease, while the patients were under observation.

Of the *nine* cases of *Typhoid*, excluding the fatal cases, the same was true of *five*.

Of the *twenty-eight* cases of *Typhus* characterized by perspiration, it took place at, or near the date of convalescence in *eleven;* and at other periods of the disease, not being speedily followed by a favorable termination of the febrile career, in *seventeen.*

Of the *five* cases of *Typhoid* characterized by perspiration, it took place at, or near the date of convalescence in not a single instance, in all the cases not being speedily followed by a favorable termination of the febrile career.

According to these results, then, the occurrence of moisture or sweating in the course of Continued Fever, considered abstractedly, cannot be considered an indication that convalescence is near at hand. The balance of probabilities, in fact, is against such an anticipation.

Directing attention now to the fatal cases, of the *five* cases of *Typhus* ending fatally, perspiration occurred toward the close of life in *two*, and at periods in the disease more or less remote from the close, in *three.*

Of the *five* fatal cases of *Typhoid,* perspiration occurred toward the close of life in *three*, and at other periods in *two.* Thus, in one half the cases of either type ending fatally, perspiration took place irrespective of its occurrence at, or near the time of death.

The foregoing results, it will be perceived on reference to the former Reports, accord, in a striking manner, with those developed by the previous analyses. The views therefore, as set forth in the quotation from the Second Report, respecting the importance to be attached to the occurrence of perspiration in the progress of Continued Fever, and to the efforts to produce this symptom by therapeutical means, are sustained, provided the reasoning therein involved be legitimate.

Another mode of interrogating the facts contained in the cases respecting the influence of perspiration on the progress of the disease, is to examine the symptoms immediately preceding, and following its occurrence. It may be supposed that perspiration generally accompanies an abatement of the severity of the disease, although it does not in the majority of instances herald convalescence. We will direct a little attention to this point. And in order to determine the condition of the patients prior, and subsequent to the occurrence of perspiration, it will probably answer to ascertain the relative frequency of the pulse at these times. The pulse may be considered a thermometer of the grade of severity of the disease sufficiently for the present object. I will give the enumerations of the pulse, in several cases, on the day previous to the occurrence of perspiration, the day on which the perspiration was noted, and the subsequent day. In the following table the instances in which perspiration occurred at periods of the disease more or less remote from the date of convalescence, will of course only be selected. In some cases perspiration continued for several days in succession, or was frequently repeated. These cases will be rejected. Fatal cases are also excluded:

| Pulse day before Perspiration. | | | Day of Perspiration. | Subsequent day. |
|---|---|---|---|---|
| No. 1. | *Typhus*, | 120. | 120 A. M. 116 P. M. | 100. |
| No. 2. | " | 108. | 104. | 104. |
| No. 3. | " | 132. | 124. | 118. |
| No. 4. | " | 108. | 100. | 88. |
| No. 5. | " | 128. | 128. | 120. |
| No. 6. | " | 156. | 134. | 128. |
| No. 7. | " | 110. | 120. | 104. |
| No. 8. | " | 106. | 124. | 136. |
| No. 9. | " | 90. | 100. | 108. |
| No. 10. | " | 111. | 108. | 98. |
| No. 11. | " | not noted. | 120. | 120. |
| No. 12. | " | 120. | 112. | 120. |
| No. 13. | " | 112. | 138. | 112. |
| No. 14. | " | not noted, day of admission. | 100. | 96. |
| No. 15. | " | 132. | *126 and 116. | 118. |
| No. 16. | *Typhoid*, | 132. | 100. | 100. |

* Perspired two consecutive days.

The results displayed in the foregoing table, obtained after the preceding portion of this section was written, are interesting, and, I confess, to me unexpected. In this point of view, perspiration appears either to occasion, or signify, an improvement, in so far as the pulse is a criterion of the latter.

Finding no grounds, numerically, for the opinion that this symptom is generally the precursor of convalescence, it was natural to presume that it would be found to possess small importance as a means, or a sign of improvement. It would appear, however, that in a very large proportion of instances, it is followed by more or less diminution in the grade of severity of the disease. This being the case, the views which have been expressed respecting the probable inutility of diaphoretics may require to be modified, since it is manifestly a legitimate object of therapeutics to endeavor to abate the intensity of disease when we are unable either to arrest it, or abridge its duration.*

In several of the cases ending in recovery in this collection, moisture, or sweating more or less copious, occurred several times during the febrile career. It may be worth while to inquire respecting the gravity of the disease in the cases thus characterized. With reference to this inquiry, I will throw into a tabular form the facts contained in the histories of these cases, relating to—*first*, the number of days on which either moisture or free sweating occurred; *second*, the mean frequency of the pulse during the febrile career; *third*, the duration of the disease from the time of taking to bed to convalescence, and *fourth*, the number of days the patients were under observation.

| Cases. | No. of days on which moisture or sweating was noted. | Mean frequency of Pulse. | Duration of Disease. | Number of days under observation. |
|---|---|---|---|---|
| 1. *Typhus.* | 5. | 112. | 13 days. | 9. |
| 2. " | 5. | 107. | 12 " | 12. |
| 3. " | 7. | 108. | 13 " | 13. |
| 4. " | 4. | 100. | 12 " | 12. |
| 5. " | 5. | 94. | 14 " | 11. |
| 6. *Typhoid.* | 8. | 89. | 16 " | 16. |
| 7. " | 5. | 80. | 16 " | 16. |
| 8. " | 3. | 80. | 23 " | 16. |

The results exhibited in the foregoing table show a grade of severity, as exhibited by the pulse and duration, somewhat less than the average in all the cases characterized by the more or less frequent recurrence of perspiration, with a single exception, viz., the first case in which the mean frequency of the pulse was 112. The inference, therefore, from the cases now analyzed,

* The reader will observe the table did not embrace cases in which perspiration occurred for several days in succession, or was frequently repeated.

would be that, other things being equal, the repeated occurrence of perspiration is generally associated with mildness of the disease. This inference, however, is by no means to be adopted as a law of Continued Fever, for, in the first place, the data are too few for such a deduction, and, in the second place, it is to be considered that perspiration was repeated in several of the small number of cases in this collection which proved fatal. The facts with respect to the latter point are as follows: — Of the *ten fatal* cases of both types, perspiration occurred more than once in *four*. In one case it was noted on *five* days; in one, *three* days; and in *two* cases, *two* days.

### SECTION TENTH.

*Symptoms referable to the Genito-urinary System.*

Notwithstanding the views expressed in the Second Report, of the importance of recording examinations of the urine in cases of Continued Fever, the histories that, in the mean time, I have collected, contain very little relating to this subject. The few observations that were made, I will give as noted in the cases respectively.

*Typhus.*

No. 1. On the *third* day the urine was acid, with a slight phosphatic deposit, and the sp. gr., 1.016.

No. 2. On the *first* day the sp. gr. was 1.023, and there was a slight trace of albumen.

No. 3. On the *second* day, the urine contained a small quantity of albumen. It deposited on cooling the urate of ammonia. On the *twelfth* day, sp. gr. 1.023.

No. 4. On the *second* day, sp. gr. 1.026. There was a considerable deposit of the urate of ammonia, and a trace of albumen. On the *fourth* day, sp. gr. 1.014, and a small quantity of albumen.

No. 5. On the *tenth* day tne sp. gr. was 1.026; it deposited urate of ammonia, and exhibited a trace of albumen.

No. 6. On the *fourteenth* day, sp. gr. 1.0–5, and a trace of albumen.

No. 7. On the *sixth* day, deposited urate of ammonia. No other change.

No. 8. On the *sixth* day, sp. gr. 1.022, a small deposit of urate of ammonia, and a trace of albumen. On the *twenty-first* day (three days before convalescence) sp. gr. 1.023, and normal.

No. 9. On the *sixth* day, sp. gr. 1.014, and a small quantity of albumen.

*Typhoid.*

No. 10. On the *ninth* day, sp. gr. 1.026, deposite of urate of ammonia.

*Twelfth* day (four days before convalescence) deposited urate of ammonia. Day after convalescence, sp. gr. 1.019.

These isolated observations are of course insufficient for analytical examination. They show that urate of ammonia is apt to be deposited at different periods of the febrile career, and that the urine frequently contains a small quantity of albumen. If a conjecture were to be indulged respecting the latter, it might be attributed to a certain degree of congestion, which it is probable exists internally, as well as upon the surface of the body, and from which the kidneys would not be likely to be exempt.

The facts relating to the specific gravity have little or no value, inasmuch as the quantity of the secretion was not ascertained.

### SECTION ELEVENTH.

### *Duration of the disease. Sequelæ. Mode of dying. Fatality.*

*Duration.* In order better to compare the two types with respect to duration, I will examine the facts pertaining to each type under a separate head, giving, in this Report, results without details. The duration from two points of view only will be considered, viz., from the date of taking to the bed, and from the time of admission into hospital, to the day of convalescence, or to the day of death.

The duration of the access has already been considered in another section. It would be useless to determine the duration in the cases not fatal to the time of discharge from hospital, as this was too often affected by circumstances entirely foreign to the disease.

*Typhus.* The data for determining the duration from the date of taking to the bed, to the day on which convalescence was pronounced, are contained in the histories of *thirty-four* cases. The mean duration is *fifteen* 8–17 days. The maximum of duration, in this point of view, was *twenty-six* days. The minimum duration was *nine* days.

The duration from the time of admission into hospital to the day of convalescence is ascertained in *twenty-five* cases.* The mean duration is *eleven* 9 – 25 days. The maximum duration, in this point of view, was *twenty* days, and the minimum duration, *six*.

*Typhoid.* The data for determining the duration from the time of taking to the bed to the day of convalescence, are contained in the histories of *six* cases. The mean duration is *eighteen* 1–3 days. The maximum duration was *twenty-three* days. The minimum duration, *eleven* days.

* The remainder of the patients were inmates of the hospital when attacked.

The duration from the time of admission into hospital to the day of convalescence, is determinable in *seven* cases. The mean duration is *thirteen* days. The maximum duration was *sixteen* days. The minimum duration, *nine* days.

These results approximate closely to those developed by the first analysis, differing, as did the latter, from those afforded by the second.

They show a shorter mean duration in *Typhus*, dating both from the time of taking to the bed, and admission into hospital.

The cases of *Typhoid* approximated, individually, nearer the mean duration than those of *Typhus*, the latter type affording the greater maximum, as well as minimum duration.

Directing attention, now, to the duration of the *fatal* cases, the following are the results:

*Typhus.* Mean duration from date of taking to bed, to fatal termination, *twelve* 3–5 days. From time of admission into hospital to fatal termination, *seven* days.

Maximum duration, from taking to bed, *eighteen* days; minimum, *nine* days.

Maximum duration, from admission into hospital, *nine* days; minimum, *five* days.

*Typhoid.* Mean duration, from date of taking to bed to fatal termination, *twenty-one* 1–5 days. From time of admission into hospital, to fatal termination, *fourteen* 4 – 5 days.

Maximum duration, from taking to bed, *twenty-seven* days; minimum, *thirteen* days.

Maximum duration, from admission into hospital, *twenty* days; minimum, *twelve* days.

*Sequelæ.* A large proportion of the patients remained in hospital but a short period after convalescence. My observations relative to *sequelæ*, therefore, not extending over much space of time, are few. I will give all the facts which appear in the histories.

*Typhus.*

*Case* 1. Mild diarrhœa occurred during convalescence.

*Case* 2. Sloughing, on the nates, superficial, and to a limited extent, occurred about the time of convalescence.

*Case* 3. Several furunculi appeared shortly after convalescence.

*Case* 4. Ecthyma occurred shortly after convalescence.

*Case* 5. In this case the patient became convalescent, and sat up on the twenty-third day after the date of the attack. On that afternoon he was attacked with a severe chill which lasted an hour, during which the pulse could not be felt at the wrist. Five or six months prior to being attacked with *Typhus*, he had had Intermittent fever. The pulse became very frequent when reaction took place, numbering 148. Several dejections occurred, and muttering delirium. The next day he had another chill in the afternoon, and on the following morning perspired freely. Death occurred on the succeeding day. The record of the symptoms after the relapse occurred is rather imperfect.

*Case* 6. This case was followed by tuberculous deposit in the lungs and spleen, and death in about two months after convalescence. The patient had had *Typhoid* fever a short time before being attacked with *Typhus*, the latter disease being developed while he remained in hospital after convalescing from *Typhoid.*

*Typhoid.*

*Case* 1. Twenty-two days after the date of convalescence, the patient was attacked with Dysentery, which proved fatal.

*Case* 2. Parotitis occurred at about the time of convalescence. Reference has already been made to this case in the section relating to the digestive system.

*Relapses.* Case No. 5, (*Typhus*,) in the foregoing list, might perhaps with propriety be considered a case of *relapse of fever.* Aside from this case, relapse occcurred but in a single instance. In this respect the present collection as well as the first, presents a striking contrast to the second. In the single instance referred to, the patient was seized with headache, pain in the limbs, and febrile movement, *seven* days after the date of convalescence, having, in the mean time, been able to sit up a little. On the fourth day after the second attack, free perspiration occurred, which was renewed for several successive nights, and the patient was again shortly convalescent.

*Mode of dying. Typhus cases.*

1. Ninth day after taking to bed, by asthenia.
2. Eleventh do., and fifth after admission, by asthenia, preceded by apnœa.
3. Twelfth do., and seventh after admission, by apnœa, apoplectic coma.
4. Eighteenth do., and ninth after admission, by asthenia.
5. Thirteenth do., and seventh do., by apnœa, apoplectic coma.

*Typhoid cases.*

1. Fifteenth day after taking to bed, and twelfth after admission, by apnœa, apoplectic coma.

2. Twenty-seventh do., and twentieth do., by asthenia.
3. Thirteenth do., and twelfth do., by apnœa.
4. Twenty-fifth day do., and twelfth do., by asthenia.
5. Twenty-sixth day do., by apnœa.

*Fatality.* The number of cases that proved fatal has already been stated, viz., *five* of the cases of each type, i. e. *ten* in the whole number of cases analyzed, viz. *sixty-four.* In this enumeration the instances of death occurring subsequent to the career of fever are not included. Death took place in one case from dysentery supervening on *Typhus*; another from relapse of fever; and a third from tuberculosis. Since in each of these instances the patients had convalesced, and the issue was not due to the fever except so far as it may have predisposed to the consecutive affections, or diminished the power of resistance inherent in the organism, it would hardly be proper to consider these as cases of fever ending fatally.

As already stated, one case of *Typhus* which, the history not having been recorded, is not included in the collection, ended fatally. I refer to the death of the Sister of Charity mentioned in the Second Section. In determining the rate of mortality this should be included, which makes *eleven* of the *sixty-five* cases, proving fatal. This gives nearly a ratio of *one* death to every *six* cases, or 17 *per centum.*

This rate of mortality is less than in the two collections of cases previously analyzed. For reasons assigned in the last Report, it would be unfair to infer from this difference the superiority of the management of the cases in this collection. The treatment, as will be seen in the section devoted to that subject, was not altogether the same as heretofore, but the fluctuations in fatality at different times and places are so great as almost to preclude any positive deductions as to the success of any particular plan of management in any collection of cases. Negative conclusions, however, may be adopted with less reserve, in view of a large proportion of recoveries. In other words, the treatment under these circumstances, it may fairly be claimed, could not have been highly pernicious.

A striking point pertains to the dates of the fatal cases. Up to Jan. 3d not a single death from Continued Fever had occurred. More than half of my six months' service had then expired, and I had then treated *fourteen* cases of *Typhus, seven* cases of *Typhoid*, and *three* cases of *doubtful type*, in all *twenty-four* cases. The dates of admission of the fatal cases, respectively, were as follows: *Typhus.*—Jan. 3; Jan. 15; Jan. 15; Jan. 26; March 5. *Typhoid.*—Jan. 19; Jan. 26; Jan. 29; Feb. 18; March 11. Thus, it is perceived, *four* of the *five* recorded *Typhus* cases, and *three* of the *five*

*Typhoid* cases proving fatal, were admitted in the month of January. If, now, the reader will turn to *Section First*, he will find that during the month of January *eleven* cases of *Typhus*, and *three* of *Typhoid* were admitted, making *fourteen cases;* so that one-half of the cases received during this month proved fatal—an enormous proportion compared with the ratio of deaths to recoveries in any other month. It would hardly seem probable that this was owing merely to coincidence, and, if not, there must have existed during that month, for reasons utterly inexplicable, an unusual tendency, in cases of fever, to a fatal result. Striking differences in this respect, in different years, and at different places, are abundantly established. It is only circumscribing within narrower limits a similar fact, to show that a greater liability of death obtains during one of a series of months, in the same situation, and under the same kind of management.

The two previous analyses developed a larger ratio of fatality in the *Typhus*, than in the *Typhoid* cases. The reverse of this is true of the present collection. The proportion of deaths was considerably greater in the *Typhoid* group, being as 5—14 is to 6—42. This disparity shows that, for reasons unknown, there existed a stronger tendency to a fatal result in the *Typhoid* cases. Compared with the previous collections, the ratio of fatality, in the cases of the latter type, is much greater than heretofore, being nearly 36 per cent.; while, on the other hand, the ratio of fatality in the *Typhus* cases is as strikingly less, being a fraction below 14 per cent.

### SECTION TWELFTH.

*Examinations after death.*

I shall restrict myself, in this Section, to the appearances presented in the intestinal canal, more especially the small intestine. The appearances in the latter situation are interesting in consequence of their bearing on the question of the identity or non-identity of *Typhus* and *Typhoid* fever. My reasons for taking this limited view of the post mortem examinations are, *first*, it is with reference to the question just stated that the contribution of facts is, in a special manner, desirable; and, *second*, for the reasons given in the previous Reports, the dissections did not generally embrace an inspection of all the important parts of the body. Intestinal lesions were always sought for with care, and the appearances recorded, but in most of the cases the condition of some organs was not ascertained, and, except with reference to some special point of interest like that relating to the lesions stated to be characteristic of the *Typhoid* type, an autopsy is unsatisfactory which does not embrace all the facts, negative as well as positive, which the scalpel can disclose. I have already had occasion to refer to two instances of death by sudden coma, with spasmodic Inspiration, (Section Seventh,) in which the larynx was inspected in order to ascertain whether œdema, or other morbid conditions existed in that part. Any other facts noted, aside from the intestinal appearances, which shall appear to have any particular interest, will be mentioned; otherwise, for the sake of brevity, they will be omitted.

Of the *five* fatal cases of *Typhus*, examinations were made in *four* instances; and of the *five* fatal cases of *Typhoid*, examinations were made also in *four*. In addition to these I have had an opportunity of inspecting the intestinal canal and other parts of a patient who has died since the expiration of my term of service, the appearances taken in connection with the history of the case being peculiarly interesting. In another case, not embraced among the cases of death from fever, in which the patient, after sitting up, suffered a relapse which proved fatal, a post mortem examination was made. The appearances in this case, which was of the *Typhus* type, may also be included.

I will give a succinct statement of the results of these examinations of the cases respectively.

### *Typhus.*

*Case* 1. *Death by asthenia, on the* 11*th day.* Several of the Peyerian patches developed so as to be visible, with black points, presenting the *shaven beard* appearance. No enlargement of the mesenteric glands. A patch of arborescent redness in the ileum just above the coecum. Large intestine free from disease.

*Case* 2. *Death by apnœa, on the* 12*th day.* A few Peyerian patches developed so as to be visible. No enlargement of mesenteric glands.

*Case* 3. *Death on the* 18*th day, by asthenia.* Deep capilliform redness of mucous membrane lining the lower part of the ileum. A single Peyerian patch near the coecum, and solitary glands developed so as to be visible. No enlargement of mesenteric glands.

*Case* 4. *Death, bg apnœa, on the* 13*th day.* Several Peyerian patches developed so as to be visible. Mesenteric glands slightly enlarged, the largest being of the size of a small pea. Two small *Tricocephali* found in the coecum.

*Case* 5. *Death on third day after relapse of fever, the patient having convalesced from Typhus.* An account of this case is given in *Section Eleventh.* So far as the post mortem appearances are concerned, it has as much value as any of the *Typhus* cases.

The Peyerian patches presented the *shaven beard* appearance, being developed so as to be visible. Some of the solitary glands also developed.

Mesenteric glands somewhat enlarged.

Several small ulcerations in coecum.

*Typhoid.*

*Case* 1. *Death, by apoplectic Coma, on the* 15*th day.* The Peyerian patches greatly developed, projecting two or three lines above the level of the surrounding mucous membrane. This enlargement was most marked in the patches nearest the coecum, and it progressively diminished on ascending the tube. The patches did not present any sloughing, except, to a limited extent, in the patch nearest the coecum. The mesenteric glands much enlarged, some as large as filberts.

*Case* 2. *Death, by asthenia, on the* 27*th day.* Peyerian patches much diseased, those nearest the coecum presenting deep excavations exposing the fibres of the muscular tunic. On ascending the tube, in some of the patches partial sloughing of the glands and morbid deposit had occurred, while in those still higher in the tube the morbid deposit remained, i. e. the patches were salient, and not ulcerated.

Mesenteric glands moderately enlarged.

*Case* 3. *Death, by asthenia, on the* 25*th day.* Peyerian patches extensively ulcerated, a large proportion presenting excavations, the morbid deposit and the glandular follicles having sloughed away.

Mesenteric glands much diseased.

Three tricocephali in the coecum.

*Case* 4. *Death on about the* 26*th day, by apnœa.* Peyerian patches developed, and those nearest the coecum presenting excavations. The solitary glands enlarged. Mesenteric glands enlarged.

*Case* 5. *Death fifty-three days after convalescence from Typhus, the patient having previously had Typhoid, being attacked while at the hospital with the former type shortly after convalescence from the latter.* To this very interesting case I have already had occasion to advert, (Section Sixth.) The patient entered the hospital with *Typhoid* fever, Dec. 27th. He had then been confined to the bed seven days. He was convalescent Jan. 11th, (sixteen days.) On February 7th he took to the bed with *Typhus*, and was convalescent from the latter on the 23d of February, (seventeen days.) Death occurred on the 15th of April, fifty-three days after the date of convalescence from *Typhus*, and ninety-six days from the date of convalescence from *Typhoid.* He presented successively the characteristic symptoms of each type of Continued Fever, including the eruptions. The following post mortem appearances were observed: The *lungs* contained numerous disseminated tubercles, and some masses of about the size of filberts. The tuberculous deposit was most abundant in the right lung. There were no excavations.

The surface of the *spleen* was studded with tubercles of pretty uniform size, about as large as small shot. Being reserved, in order that its external appearance might be copied, it was not examined internally.

The peritoneal surface over the liver presented a space of about the size of the palm of the hand, which was thickly covered with a granular deposit, apparently of tubercle.

The small intestine presented near the coecum an oval space presumed to be the site of a Peyerian patch, of a slate color, without any black points, not depressed, nor salient. The dark color caused the patch to be well defined, contrasting with the pale color of the surrounding mucous membrane. The mucous membrane over this patch was somewhat softened. Upward the same discoloration was observed in several patches, but less in degree. Numerous small oval excavations existed in these patches, extending below the mucous coat, not presenting a granulated or ulcerated aspect, but apparently covered with a delicate, serous like membrane. Several similar depressions, also, existed in the mucous membrane in the neighborhood of the Peyerian patches. Some twenty or more of these excavations were counted, being less numerous and more superficial in proportion to the distance from the coecum.

The mesenteric glands were enlarged, some being as large as a small sized bean.

The appearances in the foregoing case (No. 5) are interesting from the fact that they were the remnants of the characteristic lesions of *Typhoid ninety-six days* after convalescence from that type of fever, the patient having, in the mean time, passed through *Typhus.*

In the two former Reports the appearances found after death were submitted without comment. The few remarks which I shall offer on the subject now, will be based on the results of the examinations in fatal cases contained in all the three collections. My remarks, as already stated, will be limited to the appearances found in the small intestine. What are the conclusions to be drawn from these data as respects the appearances which distinguish the two types from each other? It is chiefly with reference to this inquiry that I propose to review the facts which I have recorded.

The three Reports contain an account of the appearances of the small intestine in *twenty-two* cases. Of these twenty-two cases, *thirteen* were of the *Typhoid* type, *eight* were of the *Typhus* type, and in one case the patient had lately passed through both types. In each of these cases the diagnosis was made during life, and based on evidence deemed unequivocal. In nearly all the cases the distinctive symptoms embraced the peculiarities of the eruption. There can be no room for doubt that in every instance the *ante mortem* history, exclusive of the *post mortem* appearances, fully warranted the determination of type. I am particular to make this statement, for it is obvious, when we are prosecuting investigations in order to ascertain what lesions distinguish either type from the other, it is necessary to settle positively on the diagnosis in individual cases, irrespective of the lesions of which we are in search. In other words, with reference to the question which has just been propounded, the post mortem appearances should not enter into the diagnosis. On this point the reader is referred to some remarks contained in the supplement to the two first Reports, under the heading of the "*Identity of Typhus and Typhoid fever.*"

What are the morbid appearances that have been found in the *Typhus* cases? In each of the *seven* examinations in cases of this type, the Peyerian patches have been somewhat changed, the alteration consisting apparently of a *slight* degree of hypertrophy, causing their development so as to render them visible. In some instances the patches have been studded with black points, giving rise to what has been called the *shaven beard* appearance. Occasionally the solitary glands have exhibited the same kind of development, and the glandular bodies of the mesentery have been *slightly* enlarged. In no case of the *Typhus* type have the appearances exceeded those just described, but more or less of these appearances have been uniformly observed. Both these facts are important to be borne in mind. These results prove sufficiently the incorrectness of the statement that the Peyerian patches and mesenteric glands are entirely unaffected in *Typhus.* So far as these results go, they show, on the contrary, that the Peyerian follicles are uniformly affected, but they contain proof only of a certain amount of morbid change, consisting, judged by the gross appearances, simply of a slight degree of abnormal development or hypertrophy.

On the other hand, what are the morbid appearances in the *Typhoid* cases? In each of the instances of this type the Peyerian patches exhibited a *notable degree* of alteration, the change not consisting in merely a slight development, but in great enlargement, causing the patches to project several lines above the level of the surrounding mucous membrane, or in excavations involving more or less destruction of the mucous coat and follicles, the surface of these excavations sometimes having a granular, ulcerated aspect, and in other instances covered by a thin, transparent serous-like tissue. Generally, the solitary glands were also the seat of similar excavations, or of great enlargement. The mesenteric glands, also, were invariably increased in size to a much greater extent than in the *Typhoid* cases, in some instances being quite large. To be more precise with respect to the *Typhoid* type, in *three* of the *thirteen* cases the Peyerian patches were notably enlarged, without excavations, and in the remaining *ten* cases the latter, with more or less enlargement, existed.

These appearances show, in the *Typhoid* cases, a morbid deposit in the Peyerian patches taking place early in the disease in considerable abundance, and in the *Typhus* cases simply a tumefaction. They show, at a more advanced stage of the *Typhoid* type, a sloughing of the enlarged glands with the morbid deposit and the mucous covering, leaving ulcerated excavations extending to the muscular tunic, sometimes leading to rupture and perforation; and, at a later stage, more or less advance toward cicatrization and filling up of these excavations; while, in the *Typhus* cases, in no instance were these processes of ulceration and sloughing apparent.

Although, therefore, the Peyerian patches are not wholly unaffected in *Typhus*, the changes, when compared with those that obtain in *Typhoid*, are quite insignificant. The contrast, as remarked in another place, is scarcely less striking than if the follicles in *Typhus* had remained invisible. The difference in the appearances is as great as that, for example, which exists, on the external surface of the body, between the efflorescence of measles, and the pustular eruption of small pox. The force of the difference in its bearing on the question of the identity or non-identity of the two types, cannot be considered to be much impaired by the fact that the Peyerian patches are frequently, if not generally changed in a slight degree in *Typhus*.

It seems to me more important to dwell on these points with some emphasis, because an idea appears to be very generally entertained that if the Peyerian patches are found to be affected in ever so slight degree in *Typhus*, its non-identity with *Typhoid* is disproved. But this conclusion assuredly does not follow, if it be true that the affection of these parts in *Typhoid* is quite different in character, as well as degree, from that in *Typhus*. Moreover, observers are likely to be confused, and distrustful in verifying the differential diagnosis by the intestinal appearances, in consequence of the erroneous idea just mentioned.

As respects the intestinal lesions of *Typhoid* fever, several points of interest remain to be studied. The nature of the *typhous* material, or deposit, does not appear to have been ascertained. It has been compared to the tuberculous deposit, and it appears to pass through changes somewhat analogous to the evacuation of tuberculous material into the bronchial tubes.

The Peyerian patches are frequently destroyed by the sloughing process. Are they afterward reproduced? What is the condition of these parts at a period considerably remote from the time of convalescence, after the processes of restoration are completed? Do the Peyerian patches *invariably* present the notable degree of alteration found in my examinations in *Typhoid*, and, in what proportion of cases, are they wholly unaffected in *Typhus?* These, and other inquiries which suggest themselves in this connection, afford interesting fields of research.

A point of interest, in conclusion, relates to the history of the eruption in the cases in which examinations were made after death. Directing attention to the cases entering into the present collection, an eruption existed in *two* of the four cases of *Typhus*, and in *all* the four cases of *Typhoid.* In one of the *Typhus* cases the eruption had mixed characters, i. e., a combination of the *Typhus* and *Typhoid* eruption. In one of the *Typhoid* cases, only *four* "rose spots" were counted, and in another case only *five.* The two last mentioned cases show that the intestinal lesions bear no relation of proportion to the abundance of the eruption.

### SECTION THIRTEENTH.

### *Treatment.*

The cases in this collection were treated with greater simplicity, but, in some respects, more efficiently than those heretofore analyzed. The first object in the management, of course, was to direct those measures which, in the opinion of the reporter, would most conduce to the welfare of the patients, and afford the best chance of recovery. It is quite superfluous to add, that this object must ever be paramount to every other in the minds of conscientious practitioners. Objects, however, of a scientific character, may properly, and in fact should, whenever practicable, be superadded — objects relating to self improvement, and the collection of facts by which, it is to be hoped, science may be enriched, and the resources of our art enlarged. In the latter point of view, two ideas were prominent in my mind during the time the cases were in progress. One of these ideas was to prescribe medicinal remedies only when clear and definite therapeutical indications were recognized, adopting, as heretofore, the expectant method. Another idea was to meet the indications which appeared to present themselves, directly and efficiently, employing for this end the fewest and simplest means adequate thereto. These ideas, it may be said, so far from possessing any novelty, are those upon which a rational practice is usually based. Without stopping to engage in any discussion on this topic, I will merely remark, that reflection and observation, as it seems to me, must convince us that every one is open to more or less self-deception on this score. So difficult is it to repress the tendency to resort to therapeutical interference, that there are few, if any, acute affections concerning which it is not still a desideratum to know what would be the consequence of permitting them to run their course uninfluenced by medication. All must admit the importance of this knowledge as a point of departure for rational principles of management. I have no reluctance to confess a desire to observe the progress of Continued Fever divested of all extrinsic influences save those which, considering my views of what was due to the interests of the patient, I should not be justified in withholding. The same tendency which stands in the way of refraining from all medication, even when it might be better for the patient, as well as more conducive to scientific knowledge, also leads to the multiplication of different remedies for the attainment of a common end. The unfavorable effect of the latter upon medical observations is two fold. It is obviously difficult to assign to each remedy its proportionate degree of efficacy when several are employed conjunctively. This is one evil. Another is, that in distributing our expectations of benefit among a variety of means, we are apt to attach undue importance to some, or to their collective operation, and thus employ too sparingly those which are the most efficient, and upon which it were better that the main reliance should be placed. Having aimed to have these considerations exert, practically, their full effect, the treatment of these cases, as already remarked, was even more simple than that pursued during the two previous years; and I shall be better able to submit a summary of the facts pertaining to this, certainly not the least interesting or important part of the history. In endeavoring to give the reader an account of the kind and amount of medication practiced, the most convenient method will be to consider the different remedies employed, under separate heads. I will, therefore, examine the records of the cases with reference to the extent to which different articles of the materia medica were prescribed.

In two cases, both of the *Typhus* type, no medicinal remedies whatever, were prescribed. The disease passed through its career favorably, sanitary measures being alone directed. In all other instances some remedies were employed.

*Laxatives.* Laxatives were prescribed in several cases in which no dejection had taken place for several successive days. In no instance was an active cathartic administered. The laxative measures consisted either of castor oil taken by the mouth in the dose of half an ounce, or in simple enemas. The oil was prescribed, I find, in *ten* cases. *Nine* of these ten cases were of the *Typhus* type. In every case but one, oil was given once only during the course of the disease. In the case furnishing the single exception to this statement, the oil was repeated on the following day. It was given in nearly every instance early in the disease, usually at the time the patients entered the hospital.

A single enema was prescribed in *three* cases, all of the *Typhus* type.

In one case in which castor oil was given, and in one of the cases in which an enema was resorted to, the dejections which followed were so frequent as to call for remedies to restrain them, which, in both instances, at once succeeded. The same was true of another case in which the patient had taken some cathartic just before entering the hospital.

In *two* instances (*Typhus*) no medicinal remedy was prescribed in addition to a single dose of oil; and in *one* case a simple enema constituted the sole treatment.

*Anodynes.* Anodynes were employed in a few cases to restrain diarrhœa, and the undue operation of laxative remedies. The tincture of opium was used for this end, taken either by the mouth, or by enema. It was prescribed in *five* cases of *Typhus*, and in *eight* cases of *Typhoid.* The proportion of the instances in the latter type, it will be perceived, is very considerably largest. In all these cases it was employed to a very limited extent, and frequently only once during the course of the disease.

In treating cases of fever, heretofore, I had been accustomed, very generally, to prescribe opium, or morphia, with a view to relieve mental aberration, and promote sleep. I have supposed that an anodyne influence was salutary even when vigilance or delirium were not prominent symptoms. In this collection of cases, however, I resorted to remedies of this class, except to restrain too frequent dejections, in but a very few instances. Morphia was prescribed in only *two* cases of *Typhus*, and in one of these it was given to allay cough. In *one* case of *Typhoid*, Dover's powder entered into the treatment.

My sense of the necessity of Anodynes, save for the object first stated, was lessened by the observation of cases in which they were omitted. Had it been otherwise, they would have been given more frequently.

*Astringents.* I gave the *tannic acid* in *one* case of *Typhoid* in which diarrhœa called for restraining measures. This was the only instance in which any remedies of the astringent class were employed.

*Sedatives.* The only articles employed which will fall under this class are *tartarized antimony and potassa*, and *camphor*. The former was prescribed in *three* cases of *Typhus*, and in *one* case of *Typhoid.* In the *Typhus* cases it was given to relieve delirium, in doses of gr. 1–16. In the *Typhoid* case it was given with reference to pneumonitis which existed as a complication. In one of the *Typhus* cases the antimony constituted the sole medicinal treatment.

*Camphor* was given in *three* cases of *Typhus* for ataxic symptoms, and in no case of *Typhoid.*

*Ammonia.* The carbonate of ammonia was given whenever there was a marked failure in the forces carrying the circulation. It was prescribed in all the cases ending fatally by asthenia. It entered into the treatment of *six* cases of *Typhus*, and *four* cases of *Typhoid.*

*Quinia.* Quinia was prescribed in doses of *three* grains, three times daily, and continued for several days in *four* cases of *Typhus.* In each of these cases it was given from the commencement of the febrile career. It was given in *one* case of *Typhoid.*

*Huxam's tincture* was prescribed in *one* case.

*Spiritus ether nitrosus.* This remedy was given in *two* instances, once for some difficulty in urination, and in the other case the indication doos not appear in the history.

*Vesication.* Blisters to the nape of the neck were resorted to whenever spasmodic inspiration was observed, or a tendency to coma. It entered into the treatment of all cases which ended fatally by apnœa. The employment of blisters was limited to the above indication, save that in one case they were applied behind the ears for external otitis. They were applied to the neck in *nine* cases of *Typhus*, and in *four* cases of *Typhoid.*

*Sinapisms and stimulating liniments.* These were occasionally employed in cases complicated with pneumonitis, and when coma existed, or was threatened.

*Diffusible stimulus.* The only form of diffusible stimulus employed, save in one or two instances, was *brandy.* This remedy entered into the management of the great majority of the cases of both types. It was omitted in but *five* cases of *Typhus,* and *two* of *Typhoid.* In these cases the disease was mild. Two of them were those which received no medicinal treatment. In one the treatment consisted only of a single enema, and in another of antimony in doses of a sixteenth of a grain.

In several of the *Typhus* cases (in all *nine*) brandy constituted the sole remedial agent employed.

The mode of administration, in all save two cases in which milk punch and egg nog were given, was with cold or warm water, from two to three parts of the latter, and, occasionally, the addition of sugar. The quantity given at a dose was generally half an ounce, or a tablespoonful. In some instances this quantity was increased to an ounce. The dose was repeated at intervals varying from half an hour to four hours. The shortness of the intervals corresponded with the apparent urgency of the indications, and the apparent effect. The frequency with which the doses were repeated varied very considerably in individual cases, and at different periods of the febrile career in the same case. I commenced its use, in a large proportion of the cases, early in the disease, frequently at the time the patients first came under observation, and it was usually continued pretty steadily throughout the disease.

Prostration, coolness of the surface, feebleness, and, more especially, notable frequency of the pulse were regarded as indicating its free administration. But it was given in many cases in which these symptoms were not particularly prominent, the object being to forestall their occurrence by sustaining the vital forces.

I have thus given a brief statement of the medicinal treatment. The dietetical and sanitary management was deemed not less important than that pertaining to the exhibition of remedies. In every instance nutriment was systematically administered; the forms being the same as heretofore, viz., essence of beef and milk porridge. These articles of nourishment were given at short intervals during the course of the disease, without waiting for an expression of, or consulting the wishes of the patients, and sometimes against their inclinations. Attention to cleanliness, ablutions, and ventilation, were attended to so far as circumstances permitted. Owing to the hospital being more crowded than hitherto, the sanitary conditions were perhaps somewhat less favorable than during the previous years. The larger number of patients also rendered it more difficult to bestow always the same vigilant attention.

I have not mentioned any efforts to cut short the disease. The only measures resorted to for this end was the *wet sheet*, which was applied in *two* cases of the *Typhoid* type. In one of these cases it was applied on the *third* day after admission, and the *fifth* of the febrile career. The fever continued, convalescence being pronounced on the *nineteenth* day.

In the other case it was resorted to on the second day after admission, and the fifth of the febrile career. The progress of the disease was unaffected, the patient being convalescent on the *sixteenth* day. In this case a relapse of fever occurred a few days after the date of convalescence.

It is at once obvious that in presenting the foregoing summary of the treatment pursued in this collection of cases, the object is not to furnish any proof of the effects, either immediate or remote, apparently due to each of the different measures in the cases individually. In order to form any estimate of these results it would be necessary to consider the details of the treatment in each case, together with the symptoms from day to day during the course of the disease. In other words, the histories of all the cases should be given at length. The enumerations that have been given only afford a *coup d'oeil* of the treatment which the cases received collectively, the remedies employed being so few, that, without occupying much space, it has been practicable to present a statement of facts which it is presumed will be rather more satisfactory than mere general statements.

The reader will perceive that the medicinal remedy upon which most dependence was placed was brandy. In this, with the administration of nutriment, consisted most of whatever efficiency belongs to the management. The other remedies, for the most part, were secondary in importance; and it is more than probable that the treatment might have been more uniformly restricted to the use of diffusible stimulus without affecting materially the results. That is to say, some of the remedies which were prescribed in a small proportion of cases, did not possess enough potency for much importance to be attached to them.

What conclusions are to be drawn from the management of these cases? I am half disposed to leave this question wholly with the reader. I shall devote to it but a very few remarks. We are to regard this collection of cases as exemplifying, therapeutically, the treatment of *Typhus* and *Typhoid* fever mainly by stimulants and nourishment. The rate of mortality certainly was not large. It is probably below the average of cases of the same description under analogous circumstances, irrespective of remedies, or under different plans of management. I am not disposed, however, to deduce from this fact any inferences as to the positive merits of the mode of treatment. It is well known that these forms of fever exhibit at different times and places widely different tendencies to a fatal result. Statistics may be referred to, showing a small rate of mortality, in which stimulants and a nutritious diet did not form a prominent feature of management. All that can be claimed in behalf of the treatment on the ground of the small fatality is, that it did not exert an unfavorable effect. In making this admission I would not be understood to mean that I attach only a negative value to the measures pursued. I cannot but entertain the conviction that these measures did exert more or less real efficacy. If I had thought otherwise, I should assuredly have withheld them to a greater or less extent. Attaching to them considerable importance, I could not conscientiously deprive the patients of the advantage which I supposed was to be therefrom derived. This is, however, an *opinion*, which is quite another thing than a truth demonstratively or logically established.

The probable influence on the rate of mortality in a collection of cases is, however, not the only, nor the most reliable source of information respecting the efficacy of remedies. We estimate their influence, in the formation of our opinions by experience, by studying their immediate effects in individual cases. This kind of observation has led me to attach considerable importance to the employment of diffusible stimulants, in conjunction with alimentation, in cases of fever. I cannot, as it seems to me, be deceived in the fact that the symptoms are frequently improved thereby, and that the condition of patients frequently deteriorates when these measures are withheld. I have in several instances in this, as in the collection of cases before recorded, observed the pulse to fall in frequency, the mind become more clear, etc., after stimulants, with nutriment, have been administered, or the quantity been increased, when it was hardly conceivable that this connection of events was one of coincidence merely. I have sometimes withheld these measures, or diminished their use, for a short time, in order to watch the result, and found their usefulness apparently verified in that way. In one of the *Typhus* cases in this collection, presenting the greatest degree of gravity, the pulse being nearly or quite 140 per minute for several successive days, I was led to try this experiment under the suspicion that the great acceleration of the pulse might possibly be due, in a measure, to over stimulation. In that instance, after the discontinuance of an ounce of brandy every half hour, for a few hours, the pulse rose from 140 to 150 and again fell to 140 on resuming the stimulus in the doses previously given. The patient finally recovered. It was frequently remarked that the pulse was more frequent, and other symptoms more unfavorable in the morning, when, owing to the greater difficulty of administering stimulants and nutriment with the same regularity during the night as during the day time, these measures had been less efficiently carried out; and I cannot but think that the happy issue of some cases was due to the vigilance with which the patients were watched at night as well as day, the quantity of stimulus being graduated carefully by the immediate effect on the pulse and other symptoms.

With respect to the uniformity with which these sustaining measures should be pursued in cases of fever, and, in this point of view, the degree of importance which belongs to them, my impressions are not so definite. I am by no means prepared to assert that they could not have been dispensed with in some cases, or employed less freely, without compromising the safety and welfare of the patients. Continued observations relative to these, and other points in therapeutics, are desirable. A combination of *negative* deductions from the analyses of different collections of cases, may, in the end, settle the principles which should govern the treatment of Continued Fever more *positively* than comports with our present knowledge of the subject.

### SECTION FOURTEENTH.

### *Cases of Doubtful Type.*

In the foregoing Sections I have made no reference to the facts contained in the histories of the cases in which the type of the disease was not determined. I have thought it preferable in concluding this Report to devote to the cases of *doubtful type* a distinct Section. They will require but a brief consideration. I shall examine them only with reference to the subject of *diagnosis.* It is in this point of view chiefly that they possess interest. The question naturally arises—'Is it true that the type of a certain portion of the cases in each collection is indeterminate?' If it be difficult or impossible to assign to 8–64 or $\frac{1}{8}$ of the cases falling under our observation their proper situation in either the *Typhus* or *Typhoid* groups, does not, it may be asked, this fact militate against the doctrine that Continued Fever is resolvable into these two forms? I propose to analyze the cases of *doubtful type*, in this collection, with reference to these inquiries. Let us see what are the facts pertaining to the distinctive diagnosis in the cases severally. Confining the examination to the points which distinguish the two types from each other, it will not occupy much space to present a summary of the history of each case. This I will proceed to do, appending to each case a few remarks, and afterward offering some general conclusions.

Case I. Thomas Daily, aged 11 years, Irish, recently arrived in this country. Entered hospital in the month of November. The attack was abrupt, having no period of access.

There were no manifestations of delirium.

The bowels were at first constipated, no dejection occurring for three successive days; afterward the bowels were loose, but diarrhœa was not a prominent symptom.

There was moderate meteorism, and considerable abdominal tenderness.

No eruption.

No epistaxis.

The maximum of the frequency of the pulse was 144; the mean frequency a fraction over 115.

Capillary congestion was moderate. The duration of the febrile career was about twelve days.

*Remarks.* From the foregoing assemblage of symptoms the type of the disease is presumed to have been *Typhoid.* This opinion is mainly predicated on the abdominal symptoms, which, although not present from the first, nor prominent at any time, were yet sufficiently marked. The abruptness of attack is unusual, but with respect to this, as well as other points, it is not improbable that a collection of cases of the disease occurring in young subjects would show some variation from the laws obtaining in cases of adults.

Case II.—Mary Daily, sister of No. 1, aged about eight years. Admitted in November. Attack abrupt, took at once to the bed. No manifestations of delirium. Deafness was marked.

Mild diarrhœa from the first, or, rather, looseness of the bowels.

Meteorism and tenderness is not mentioned.

Maximum of frequency of pulse, 152; mean frequency, 107.

Moderate capillary congestion during the early part of the career.

Epistaxis occurred on the third day.

Duration, fourteen days.

*Remarks.* There can scarcely be a question respecting the type in this case. It was doubtless *Typhoid.* The evidence is stronger than in case No. 1, and this being so, the fact of the relationship of the patients adds to the evidence in the first case. The history is defective in not containing statements relative to tympanites and tenderness. This was the reason, chiefly, of the case being excluded from the *Typhoid* group.

Case III.—Henry Nigelab, German, aged 40. Admitted in December. Been in America two years, and in Buffalo five weeks.

Attack was abrupt.

Delirium occurred on the third day, consisting in incoherent talking, and efforts to get out of bed. Eyes suffused. Bowels constipated, two dejections only occurring in the space of eight days. No abdominal tenderness, nor tympanites.

An indistinct eruption noted on one day.

Maximum of pulse, 128; mean frequency, 107.

Moderate capillary congestion. Epistaxis (moderate) on the fourth day.

Duration, eight days.

*Remarks.* The type of the disease was unquestionably *Typhus.* The absence of abdominal tenderness and meteorism, the constipation, the sudden attack, the age of the patient, the early delirium, the mean frequency of the pulse, constitute conclusive proof of the correctness of this opinion; and had the details of the histories been studied as fully at the preliminary examination, when the distribution of the cases were made, as now, the case would have been included in the *Typhus* group.

Case IV.—Aaron Young, English, aged 23. Admitted January. Been in this country but five days, and stated that there was fever on board the vessel in which he came over. He entered January 11th, with cough, debility, and febrile movement. He kept the bed several days, when he began to sit up, and was supposed to be convalescing from an attack of ephemeral fever. The chest was not explored at that time, but subsequently the existence of tuberculosis was determined. He was probably laboring under incipient phthisis when he entered.

On the 16th of February, (thirty-six days after date of admission) he was attacked with symptoms of fever.

He took to the bed after an access of *two* days duration.

Delirium was manifested on the third day, consisting of incoherency and efforts to get out of bed.

The bowels were rather loose, i. e. on some days two dejections occurred during the twenty-four hours.

There was moderate tympanites, but no abdominal tenderness.

On the second day after taking to the bed a copious eruption appeared, consisting of *maculæ*, dusky, not disappearing on pressure, intermingled with which were *rose spots.*

Capillary congestion moderate.

Maximum of pulse, 108; mean frequency, 95.

Slight epistaxis on the fourth day.

Duration, thirteen days.

*Remarks.* The looseness of bowels, together with the mixed eruption led to the rejection of this case, but that it was a case of *Typhus* hardly admits of doubt. This is shown by the abrupt attack, the short duration, the absence of abdominal tenderness, the early manifestations of delirium, the early appearance of the eruption, its copiousness extending to extremities as well as over the body, and the probability of the disease having been contracted by contagion in the hospital ward.

Case V.—John Jany, Irish, aged 22. Admitted in February.

Been in this country ten days.

Duration of access not determined.

No manifestations of delirium.

One dejection daily during the career of the disease, save that on one day there were two.

No tympanites, nor abdominal tenderness.

On the fourth day there was no eruption. On the fifth, sixth, and seventh days the daily record of symptoms was not made. On the ninth day there were three *rose spots* which remained for several days.

The maximum of pulse was 90; mean frequency, a fraction over 75.

Moderate capillary congestion.

Duration of febrile career fourteen days.

*Remarks.* The interruption in the daily records, and consequent imperfection of the history, was the chief reason for excluding this case. It was a case of *Typhoid* without doubt, of a very mild grade.

Case VI. — John Welch, aged 25, admitted in March.

Been in this country five years.

Entered on the *sixth* day after taking to the bed.

Access of eight days duration.

Slight manifestations of delirium, consisting only of incoherency at night.

Mild diarrhœa during the whole of the febrile career.

Moderate meteorism and slight abdominal tenderness.

Presented at the time of his admission an eruption which is described as dusky, not elevated, and the redness not disappearing on pressure. The same characters are recorded the following day. On the next day it is simply noted that the eruption remains. On the next day several *rose spots* were observed, disappearing on pressure.

Maximum of pulse, 120; mean frequency, a fraction over 104.

Moderate capillary congestion.

Epistaxis occurred early in the disease.

Duration, eighteen days.

*Remarks.* The variation in the characters of the eruption in this case renders the diagnosis doubtful. The patient, it will be perceived, did not enter until the sixth day, so that the time of the development of the eruption, and the character which it at first exhibited are not ascertained. Aside from the eruption, the symptoms clearly point to *Typhoid.* The long duration of the access and the duration of the febrile career; the abdominal symptoms; absence of delirium, and the occurrence of epistaxis, would render the discrimination sufficiently easy, were it not for the mixed characters of the eruption.

This, it will be observed, is the first of the cases of *Doubtful type* in which the facts pertaining to the history really occasion much hesitation as to the diagnosis.

CASE VII. — Charles Madden, Irish, aged 27, admitted in March. He entered hospital three weeks before being attacked with fever, with some trifling ailment which was not closely investigated. He was able to be up and about until attacked with fever.

It is not stated whether he had recently arrived in this country.

Access of four days duration.

Slight manifestations of delirium on the second night after taking to bed.

Diarrhœa, the dejections being frequent, occurred on the second and third days, but did not afterward continue. Previously there was constipation. No meteorism, nor abdominal tenderness.

An eruption appeared on the second day consisting of three or four *rose spots*. The next day there was a copious eruption, dusky, not disappearing on pressure, and intermingled a few *rose spots*.

Maximum of pulse, 114; mean frequency, 96.

Moderate capillary congestion.

Duration of disease, thirteen days.

*Remarks*. The *rose spots*, and the diarrhœa, give rise to some doubt as to the diagnosis in this case. Aside from these, the evidence of *Typhus* predominates. The absence of tympanites and tenderness over the abdomen; the early development of delirium; the appearance of the eruption early in the disease and its copiousness; the short duration of the disease and the probability of its having been contracted in the hospital, are in favor of the *Typhus* type.

CASE VIII. — George Gaylord, aged 25, English, admitted in January.

Been in America ten months, and in Buffalo eight.

A brother of this patient entered December 2th, with *Typhus*, and his case is included in the Typhus group.

Admitted on the *fifth* day after taking to the bed.

Duration of access, *three* days.

Slight incoherency from the time of admission.

Bowels constipated until the eighteenth day, when they were slightly loose.

Slight tympanites noted on one day only.

No abdominal tenderness.

Eruption abundant, presenting mixed characters. It is thus described: "Eruption copious over the body and extremities, mostly consisting of *maculæ,* not disappearing on firm pressure, rather dusky. Intermingled with these a sparse eruption of *rose spots*, larger in size, elevated, and the redness readily disappearing on pressure."

The eruption was present when admitted, and had been already observed for two days, making its first appearance as early as the third day.

Maximum of pulse, 124; mean frequency, a fraction over 111.

Moderate capillary congestion.

Slight epistaxis on the fourth day.

Duration of the disease twenty-three days.

Convalescence was retarded by pneumonitis.

*Remarks.* There is but little room for doubt in this case as to the *type*. What doubt exists is occasioned by the mixed characters of the eruption. Admitting that the eruption does present occasionally characters more or less mixed, without necessarily compromising the claims of such cases to be considered either *Typhus* or *Typhoid*, I should have been warranted, as it appears to me, in including this case in the former group. The early development of the eruption, its copiousness and extent; the short duration of of the access; the mean frequency of the pulse, and the fact that a brother, shortly before, with whom he had been associated, was admitted with clearly marked *Typhus*, render the diagnosis almost, if not quite positive.

It is sufficiently apparent from the circumstances relating to the histories of these cases, that the type of the disease, generally, was not very doubtful. In fact there were but two cases (Nos. 6 and 7,) in which much uncertainty could be considered to rest upon the diagnosis. That these few cases were included in the class of *Doubtful type*, does not, therefore, show that difficulty in practically discriminating between the two types, which the reader, without these details, might naturally have been led to infer. Their exclusion from the *Typhus* and *Typhoid* groups, was owing to the care not to include in either group a single case in which the type was in the least degree doubtful, it being deemed far better to be hypercautious in this respect, than to run any risk of error. So far as my observations go, the principles of diagnosis briefly presented in the Supplement to the Second Report, admit of a ready and positive application in all but a very small proportion of instances. The fact must be considered to have considerable significance in connection with the question as to the identity of *Typhus*, and *Typhoid* fever. At the same time, the fact is not to be overlooked that we do find, in a certain proportion of cases, a combination, to a greater or less extent, of the symptoms distinctive of either type. The latter fact applies to the eruption, as well as other events belonging to the natural history of the two forms of fever. In view of this fact, it may seem, in the minds of some, to be a rational conclusion that the two forms of Continued Fever are not so distinct but that they may be blended, developing a kind of hybrid disease, which cannot properly be considered to be either *Typhus* or *Typhoid*. This certainly opens a very interesting point of inquiry. It cannot, however, be prosecuted, with any expectation of coming to a satisfactory conclusion, by means of speculative reasoning, and my observations relative to the subject do not afford data adequate to any logical deductions.

*Note.* It is proper to state that the histories of the cases upon which the foregoing Report is based were, for the most part, not recorded by my own hand, but by Mr. J. Lewis Smith, resident medical student at the hospital, for whose faithful services I desire to express my obligations. The records were made under my daily direction and supervision.

